# Pancreatic endosonographic findings and clinical correlation in Crohn's disease

**DOI:** 10.6061/clinics/2019/e853

**Published:** 2019-05-24

**Authors:** Éverson Fernando Malluta, Fauze Maluf-Filho, André Zonetti de Arruda Leite, Carmen Lucia Ortiz-Agostinho, Iêda Nishitokukado, Adriana Ribas Andrade, Maria Laura Lacava Lordello, Fabiana Maria dos Santos, Aytan Miranda Sipahi

**Affiliations:** 1 Laboratorio de Gastroenterologia Clinica e Experimental, Hospital das Clinicas HCFMUSP, Faculdade de Medicina, Universidade de Sao Paulo, Sao Paulo, SP, BR.; 2 Departamento de Gastroenterologia, Instituto do Cancer do Estado de Sao Paulo, Sao Paulo, SP, BR.

**Keywords:** Crohn's Disease, Pancreatitis, Pancreatic Exocrine Insufficiency, Endosonography, Cholangiopancreatography

## Abstract

**OBJECTIVES:**

We aimed to evaluate the incidence of pancreatic alterations in Crohn's disease using endoscopic ultrasound (EUS) and to correlate the number of alterations with current clinical data.

**METHODS:**

Patients diagnosed with Crohn's disease (n=51) were examined using EUS, and 11 variables were analyzed. A control group consisted of patients with no history of pancreatic disease or Crohn's disease. Patients presenting with three or more alterations underwent magnetic resonance imaging (MRI). Pancreatic function was determined using a fecal elastase assay.

**RESULTS:**

Two of the 51 patients (3.9%) presented with four EUS alterations, 3 (5.9%) presented with three, 11 (21.5%) presented with two, and 13 (25.5%) presented with one; in the control group, only 16% presented with one EUS alteration (*p*<0.001). Parenchymal abnormalities accounted for 39 of the EUS findings, and ductal abnormalities accounted for 11. Pancreatic lesions were not detected by MRI. Low fecal elastase levels were observed in 4 patients, none of whom presented with significant pancreatic alterations after undergoing EUS. Ileal involvement was predictive of the number of EUS alterations.

**CONCLUSION:**

A higher incidence of pancreatic abnormalities was found in patients with Crohn's disease than in individuals in the control group. The majority of these abnormalities are related to parenchymal alterations. In this group of patients, future studies should be conducted to determine whether such morphological abnormalities could evolve to induce exocrine or endocrine pancreatic insufficiency and, if so, identify the risk factors and determine which patients should undergo EUS.

## INTRODUCTION

Chronic pancreatic involvement in patients diagnosed with Crohn's disease (CD) has been the focus of studies in recent years (1-7). Medications, primary sclerosing cholangitis, inflammatory involvement of the duodenum and autoimmune pancreatitis (AIP) are all well-established causes of pancreatitis in this population (8-12). Studies on this topic are limited and extremely heterogeneous, mainly because there is no established consensus method to study the pancreas. The prevalence of this combination (CD and pancreatitis) ranges from 1.2% to 58% depending on the population studied and the method of pancreatic evaluation employed (5,6,13-18).

*Postmortem* studies of patients diagnosed with CD that did not present with pancreatic symptoms have found pancreatic fibrosis (38% of cases) distributed in the interlobular and periductal areas, as well as acinar dilation (31% of cases) (15,19). Pancreatic lesions are believed to result from the formation of immunocomplexes and autoantibodies against the pancreas, which are known as glycoprotein 2 (GP2) –specific pancreatic autoantibodies (PAB) (1,9,20,21). Furthermore, the formation of epithelioid granulomas has been described in patients with CD (14,22). Epithelial cells of the gastrointestinal tract and pancreatic tissue may share similar target molecular or cellular structures vulnerable to injuries (23), as observed in animal studies where there was overexpression of abnormal proinflammatory hypoglycosylated mucinin, both in the colonic epithelium of patients with inflammatory bowel disease (IBD) and in the pancreatic ductal epithelium (24).

Endoscopic retrograde cholangiopancreatography (ERCP) is considered the gold standard for the study of pancreatic morphology. However, in addition to morbidity risks, this method evaluates only the pancreatic ducts, providing no information on the conditions of the pancreatic parenchyma (25,26). Endoscopic ultrasound (EUS) is more sensitive, is safer, evaluates both the parenchyma and the pancreatic ducts and correlates strongly with histological findings (27-33).

Although there is considerable agreement between EUS and ERCP when the EUS results are either normal or when they reveal moderate to severe pancreatitis, many patients have presented with mild EUS alterations but have normal ERCP results (34-37). Elderly patients, obese patients, and patients with diabetes mellitus, as well as patients who consume excessive amounts of alcohol, present with EUS alterations in the pancreas that are unrelated to disease. With the exception of these patients, studies have hypothesized that patients presenting with altered EUS but normal ERCP results are suffering from pancreatitis in its initial stages because, pathophysiologically, parenchymatous alterations precede ductal alterations in the necrosis-fibrosis sequence in the development of chronic pancreatitis (38). To increase the specificity of EUS, some authors have suggested that the pancreas should be considered normal if only one or two endoscopic criteria are met, whereas a pancreas displaying three or more of these criteria should be considered abnormal (30,31,34,39).

Exocrine pancreatic insufficiency has been reported in IBD in 18 to 80% of cases, as measured by the fecal elastase, paraminobenzoic acid (PABA), amylase, lipase meal and secretin-cerulein tests. (7,14,40). Fecal elastase is considered the gold standard among noninvasive tests of pancreatic function (41). Although the results obtained by this method are in agreement with the tests of pancreatic function that use duodenal intubation (42), it can yield false positives for intestinal diseases (because of dilution and consequent reduction in enzymatic concentration) and displays low sensitivity to mild or moderate pancreatitis (38,41,43-46).

In the present study, we sought to evaluate the incidence of pancreatic alterations identified using EUS in patients with established CD and correlate these results with factors such as duration of the disease, disease site, medication use, and level of disease activity. To correlate these morphological alterations with pancreatic exocrine function, we decided to measure fecal elastase levels. We also compared the EUS findings with magnetic resonance cholangiography (MRCP) findings. To the best of our knowledge, this study is the first to analyze pancreatic EUS abnormalities in CD patients, which is an important tool to detect preliminary alterations of the pancreas.

## MATERIALS AND METHODS

Fifty-one patients between 18 and 60 years of age who were diagnosed with CD on the basis of clinical, radiological, endoscopic, or anatomopathological criteria were selected to participate in this prospective study.

Patients previously diagnosed with pancreatitis, sclerosing cholangitis, or diabetes mellitus; patients who were obese; or patients who had previous gastrectomy or gastro-jejunal bypass were excluded, as were those who consumed more than 30 g of alcohol/day or who were active smokers (less than 5 years after quitting).

Data regarding age, gender, elapsed time since disease onset, CD activity index (47), anatomical location of the disease, extraintestinal manifestations, and surgical history, as well as prior and current use of medications, were collected. Serum levels of amylase and lipase were also determined for all of the patients studied.

### Endoscopic ultrasound

EUS was conducted by the endoscopy team at the Hospital das Clínicas using a 7.5 MHz linear transducer (GFUCT 140; Olympus, Hamburg, Germany) under propofol sedation. The pancreas was evaluated by 2placing the transducer in the duodenal bulb (for visualizing head of the pancreas), second and third part of the duodenum (uncinated process) and over the stomach (body and pancreatic tail). The diameter of the pancreatic duct was measured at the confluence of the splenic vein and the upper mesenteric vein.

**Figure 1:**
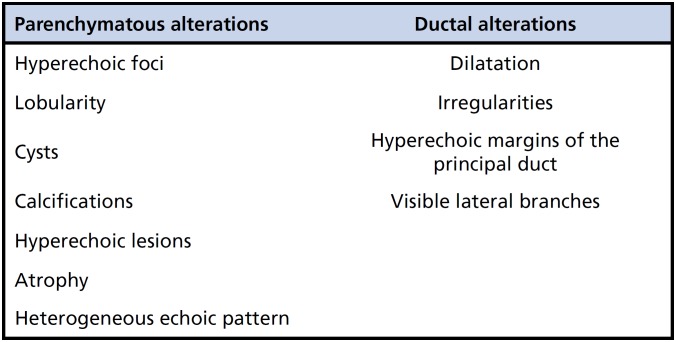
Endoscopic alterations suggestive of chronic pancreatitis.

As shown in Figure 1, pancreatic alterations identified through EUS were classified as either parenchymatous or ductal (28,29,47). Three or more of these criteria were considered abnormal. Twenty patients in the control group who did not have pancreatic disease, biliary disease, or a history of inflammatory intestinal disease also underwent EUS (EUS control group).

### Magnetic resonance cholangiography

Patients who presented with three or more EUS alterations suggestive of chronic pancreatic also underwent MRCP to determine whether there was a correlation between these two methods.

A 1.5 T magnetic resonance imaging (MRI) scanner was used (Sigma; GE Medical Systems, Milwaukee, Wisconsin, USA). The sequence carried out for each pass was as follows: axial T2-weighted fast spin-echo; axial spoiled gradient-recalled dual-echo (in-phase and out-phase); coronal single-shot fast spin-echo with fat saturation; axial spoiled gradient-recalled echo with fat saturation; and axial dynamic spoiled gradient-recalled echo (arterial and portal).

Sequences were obtained during apnea with the cooperation of the patients who had fasted for 8 h prior. No type of contrast agent or sedation was administered. The sequences were performed in three spatial planes and, occasionally, in oblique planes. Images were projected at maximum intensity and analyzed using multiplane reconstruction at a workstation.

### Fecal elastase

Pancreatic fecal elastase levels were determined using an enzyme-linked immunosorbent assay (ELISA) that used two polyclonal antibodies that recognize different epitopes at defined sequences of human pancreatic elastase according to the manufacturer´s instructions (BIOSERV Diagnostics, Rostok, Germany).

Samples were collected from 39 patients in the CD group. Samples were collected from 10 patients with chronic alcoholic pancreatitis (chronic pancreatitis group) and from 10 individuals without any clinical symptoms, radiological findings, or laboratory test results indicative of pancreatic disease (fecal elastase control group) to serve as positive and negative controls. All tests were carried out in duplicate.

### Ethical aspects

The present study was approved by the Ethics in Research committee of the University of Sao Paulo Medicine School - Hospital das Clínicas. All patients gave written informed consent.

### Statistical analysis

Statistical analysis was carried out using the Statistical Package for the Social Sciences (SPSS) program for Windows, version 10.0. Comparisons between patients with CD and the EUS control group were made using Student's *t*-test. One-way ANOVA was used for comparisons among the CD group, chronic pancreatitis group and fecal elastase control group. For analysis of the correlation between different variables in the CD group, Spearman's correlation coefficient was used. Values of *p*<0.050 were considered statistically significant.

## RESULTS

Of the 51 patients examined by EUS, 28 (56%) were female. The mean age was 38 years (range, 18–59 years). The mean elapsed time since diagnosis of the disease was 7 years (range, 1–25 years), and the mean CD activity index was 102 (range, 20-419). The majority of patients presented with ileal involvement, either isolated (47% of cases) or in combination with colonic involvement (approximately 37% of cases). In 8% of the cases, only the upper gastrointestinal tract was affected.

Approximately 40% of the patients currently presented with or had a history of enteral fistulas, and 20% presented with stenosing CD. Extraintestinal manifestations of the disease (mostly osteoarticular) were observed in 58% of the patients. With respect to current and previous medication use, 56% reported having used azathioprine (Table 1).

**Table 1:** Epidemiological features of Crohn's disease patients.

Variable		
Gender, n (%)		
	Female	28 (55)
	Male	23 (45)
Age (years), mean ± SD		38.5 ± 10.4
Years since diagnosis, mean ± SD		7.2 ± 5.7
Azathioprine use, n (%)		
	Yes	29 (57)
	No	5 (10)
	No data	17 (33)
Amylase, mean ± SD		74.7 ± 28.7
Lipase, mean ± SD		32.7 ± 11.6
Extraintestinal manifestations, n (%)		
	Yes	30 (59)
	No	16 (31)
	No data	5 (10)
Crohn's disease activity index, mean ± SD		102.0 ± 80.0
Anatomical location, n (%)		
	Ileum	23 (45)
	Colon	6 (12)
	Ileum-colon	16 (31)
	Upper gastrointestinal tract	6 (12)
	Anorectal	0 (0)
Disease presentation, n (%)		
	Stenosing	12 (23)
	Fistulizing	21 (41)
	Inflammatory	8 (16)

SD: standard deviation.

Although none of the patients presented with an elevated level of serum lipase, 9% presented with an increase in serum amylase (less than 1.5 times higher than the upper limit of normal).

### Endoscopic ultrasound

All 51 patients underwent EUS examination. According to the EUS results, 2 patients (3.9%) presented with four of the abnormalities suggestive of chronic pancreatic disease (as described in the Methods section), and three patients (5.9%) presented with three abnormalities (Table 2).

**Table 2:** Endoscopic ultrasound findings*.

EUS alterations (n)	Crohn's disease, n (%)	Controls, n (%)
4	2 (3.9)	0
3	3 (5.9)	0
2	11 (21.5)	0
1	13 (25.5)	3 (16)

* Patients with Crohn's disease *vs* controls, *p*<0.001.

EUS:, endoscopic ultrasound.

The prevalence of parenchymatous abnormalities (39 out of 51) was significantly higher than that of ductal abnormalities (11 out of 51) among patients with CD (Table 3).

**Table 3:** Endoscopic ultrasound findings of parenchymatous and ductal alterations.

Alteration	Crohn's disease (%)	Controls (%)
Parenchyma		
Hyperechoic foci	15 (35)	3 (100)
Hyperechoic lesions	11 (21)	0
Globular external margins	4 (7)	0
Lobularity	1 (2)	0
Cysts	0	0
Calcification	0	0
Atrophy	2 (3)	0
Heterogeneous echoic pattern	6 (11)	0
Ductal		
Principal duct dilatation	0	0
Secondary duct dilatation	0	0
Ductal irregularity	0	0
Hyperechoic ductal margins	11 (21)	0
Total	50 (100)	3 (100)

* Patients with Crohn's disease *vs* controls, *p*<0.001.

Of the control group, 3 patients (16%) presented with one alteration by EUS examination. The remaining control group patients presented with no abnormalities on the EUS (Table 2). The difference between the two groups was statistically significant (*p*<0.001). The only variable that correlated with the EUS data was the ileal location of the disease (*p*=0.040).

### Magnetic resonance cholangiography

The patients presenting with three or more abnormalities by EUS were examined by MCRP. One of these patients died prior to MCRP. The cause of death was unrelated to CD or to the tests that were carried out in this study.

None of the patients who were examined by MCRP presented with any abnormalities.

### Fecal elastase

The mean levels of fecal elastase (μg of elastase/g of feces) for each group were as follows: 450 μg/g for the CD group, 487 μg/g for the control group, and 125 μg/g for the chronic pancreatitis group. No significant difference was observed between the CD group and the fecal elastase control group. There was no correlation between the CD activity index and the level of fecal elastase.

Four patients (10%) presented with results suggestive of exocrine pancreatic insufficiency (a fecal elastase value <200 μg/g of feces), and 2 of these patients were classified as having severe exocrine insufficiency. For EUS, none of these 4 patients presented with any abnormalities in pancreatic morphology. In contrast, patients with three or more EUS abnormalities presented fecal elastase levels that were within the normal range. In addition, no correlation was found between the levels of fecal elastase and the epidemiological characteristics of the population.

## DISCUSSION

There is a growing number of studies linking IBD and pancreatitis, as shown in a recent review by Srinath et al. (48). The main subtypes are acute pancreatitis, chronic pancreatitis, AIP and asymptomatic abnormalities. The present study shows that 9.8% of the patients with CD presented with pancreatic involvement (three or more abnormalities) in morphological evaluations using EUS, predominantly in patients with ileal involvement. In other words, ileal involvement was predictive of the number of EUS alterations.

This finding is in line with those published in 2012 by Pavlidis et al. (21), showing that ileal inflammation may trigger the development of GP2-specific PAB in patients with CD. This protein is present on the apical surface of microfold (M) intestinal cells located in the ileum but not in colonic cells, which explains the close relation between higher positive rates in CD (almost 40%) *versus* fewer than 8% in UC.

Our findings are also in agreement with other studies on the morphological evaluation of the pancreas in patients with CD. In a retrospective study involving 255 patients with CD, the prevalence of pancreatic abnormalities in individuals presenting with no signs or symptoms of chronic pancreatitis on abdominal ultrasound, computed tomography, or ERCP was 6.3% (18). In a prospective study evaluating pancreatic morphology in patients with CD, Heikius et al. (16) used ERCP as a diagnostic tool. The authors observed abnormalities suggestive of chronic pancreatitis in 4 (8.7%) out of 46 ERCP procedures performed. In a prospective study, Barthet et al. (7) found a 30% prevalence of pancreatic insufficiency among IBD patients with no previous history of pancreatopathy and a 50% prevalence among patients with previous symptoms. In that study, patients were evaluated by pancreato-MRI and exocrine function by fecal elastase test, amylase, lipase, C-reactive protein, pancreatitis associated protein (PAP), IgG4 and PAB.

In our study, the higher prevalence of pancreatic abnormalities (9.8% of the patients presented with three or more EUS abnormalities) can be explained by differences in the morphological evaluation method. Importantly, three or more positive EUS signs are suggestive of chronic pancreatopathy instead of chronic pancreatitis. The sensitivity of EUS in evaluating the pancreatic duct and the parenchyma, which is crucial to the early detection of chronic pancreatitis, appears to be greater than that of ERCP (38,49). For the evaluation of pancreatic diseases in patients with CD, EUS is the most rational choice because pancreatic abnormalities in this situation are predominantly parenchymatous, as has been shown in anatomopathological studies (15,50). By evaluating our patients endoscopically, we found more parenchymatous abnormalities predominated than ductal abnormalities, accounting for 39 and 11 findings, respectively. Based on these results, we may presume that CD patients have pancreatic abnormalities similar to those of patients with obesity, diabetes and alcohol intake, and the vast majority are asymptomatic. However, our results are from only one observer. We could not integrate interobserver agreement into the study, which would have led to more reliable results. Additionally, the patients who had EUS alterations underwent MRCP without any contrast agent. The use of gadolinium could provide more information on minimal parenchymal changes. All patients in the study who submitted to this procedure had no abnormalities.

We identified pancreatic insufficiency in 10% of patients with CD by fecal elastase level determination, although all 4 patients who had values below the threshold of 200 μg/g did not have any ultrasonographic changes, suggesting that some patients with CD might have a functional pancreatic insufficiency not related to morphologic changes in the gland. This finding is in agreement with previous results obtained for patients with idiopathic pancreatitis (51), as well as for patients with mild or moderate pancreatitis (35,52-54). Lindstrom et al. analyzed patients with inflammatory intestinal disease and primary sclerosing cholangitis (55). The authors observed primary abnormalities in morphology rather than in pancreatic function. The fecal elastase test has an insufficient sensitivity (68–77%) and is inappropriate to demonstrate early chronic pancreatitis with a cut-off value of 200 g/g stool (45,56-58). The sensitivity of the fecal elastase test is closely related to the severity of pancreatic insufficiency (45,57).

Only four previous studies have analyzed exocrine pancreatic function in patients with CD (5,6,16,17). Among the patients studied, the proportion presenting with a decrease in pancreatic function ranged from 4.2% to 58%. Such variation occurred due to the differences among the methods used to evaluate pancreatic function. Among the studies employing invasive tests (*e.g.,* those involving duodenal intubation), one used a secretin test to evaluate pancreatic function in 54 patients, 2 (4.2%) of which presented with abnormalities (16). Angelini et al. used the secretin-cerulein test in 17 patients with CD (5). The authors observed that in the duodenal aspirate, 35% of the patients presented with a decrease in the secretion of enzymes and bicarbonate, whereas 58% presented with a decrease only in the concentration of lipase. Using the Lundh meal test, Hegnohj et al. noted a significant decrease in the concentration of amylase and lipase in the duodenal aspirate of 143 patients with CD and that the decrease was significantly greater in patients presenting with extensive ileal involvement (6). Seibold et al. used noninvasive methods (fluorescein dilaurate and fecal chymotrypsin) to evaluate pancreatic function in patients with CD (17). The authors found that the prevalence of impaired pancreatic function was approximately 15%, regardless of the method employed and that such impairment was more common in patients with autoantibodies against the exocrine pancreas.

AIP was recently described (59). AIP is characterized by serum IgG4 elevation associated with predominantly lymphoplasmocytic inflammatory infiltrate and is accompanied by pancreatic fibrosis, thereby provoking pancreatic insufficiency (60-62). AIP can be associated with inflammatory intestinal disease in 5% to 22% of cases (12,20,60,63), and an elevation of IgG4 was observed in colonic biopsies from IBD patients even without pancreatic disease (64). The most common EUS findings are a focal or diffuse increase in the size of the pancreas, together with diffusely or focally hypoechoic pancreatic parenchyma (65). In a recent study, Hoki et al. concluded that the Sahai criteria (34) for chronic pancreatitis are inadequate for the evaluation of AIP, where the average score was only 2 (66). A periductal infiltrate composed of lymphocytes and plasmocytes accompanied by inter and intralobular fibrosis is histologically observed. Atrophy of the parenchyma can also occur (60). It is possible that some of our patients had IgG4-related disease, which might alter the sonographic appearance of the organ.

In a study of the pancreas, EUS findings were correlated with the histopathological findings (29). In our study, the principal findings were hyperechoic foci (35%), hyperechoic ductal margins (21%), hyperechoic lesions (21%) and a heterogeneous echoic pattern (11%). These alterations correspond, respectively, to focal fibrosis, periductal fibrosis, bridge fibrosis and edema, characteristics that are quite prevalent in AIP.

The pancreatic alterations observed in CD are specifically due to the activation of immunocomplexes (4,8,20) since some of these patients present with high titers of circulating autoantibodies against the exocrine pancreas (17,67-69), which have been directly related to the presence of pancreatic insufficiency (17). These autoantibodies are believed to induce tropism in exocrine pancreatic acinar cells (14). Histologically, B lymphocyte aggregates are observed in the pancreatic tissue (20). Pancreatic alterations are believed to be less common and less prominent than other extraintestinal manifestations of CD because autoantigens come into contact with the immune system only outside of the pancreas (67).

## CONCLUSION

In conclusion, the present study showed that in a sample of 51 patients, 5 (9.8%) presented with three or more EUS abnormalities suggestive of chronic pancreatitis, which is significantly different than the number observed for the control group. However, none of these 5 patients presented with any clinical signs or laboratory test results (*e.g.*, fecal elastase levels) indicative of pancreatic exocrine insufficiency.

In this group of patients, future studies should be conducted to determine whether such morphological abnormalities could evolve to induce exocrine or endocrine pancreatic insufficiency and, if so, identify the risk factors and determine which patients should undergo EUS (8,10,23).
